# Self-consistent determination of the spike-train power spectrum in a neural network with sparse connectivity

**DOI:** 10.3389/fncom.2014.00104

**Published:** 2014-09-18

**Authors:** Benjamin Dummer, Stefan Wieland, Benjamin Lindner

**Affiliations:** ^1^Theory of Complex Systems and Neurophysics, Bernstein Center for Computational NeuroscienceBerlin, Germany; ^2^Department of Physics, Humboldt Universität zu BerlinBerlin, Germany

**Keywords:** neural noise, recurrent neural networks, non-Poissonian spiking, spike-train statistics, spike-train power spectrum

## Abstract

A major source of random variability in cortical networks is the quasi-random arrival of presynaptic action potentials from many other cells. In network studies as well as in the study of the response properties of single cells embedded in a network, synaptic background input is often approximated by Poissonian spike trains. However, the output statistics of the cells is in most cases far from being Poisson. This is inconsistent with the assumption of similar spike-train statistics for pre- and postsynaptic cells in a recurrent network. Here we tackle this problem for the popular class of integrate-and-fire neurons and study a self-consistent statistics of input and output spectra of neural spike trains. Instead of actually using a large network, we use an iterative scheme, in which we simulate a single neuron over several generations. In each of these generations, the neuron is stimulated with surrogate stochastic input that has a similar statistics as the output of the previous generation. For the surrogate input, we employ two distinct approximations: (i) a superposition of renewal spike trains with the same interspike interval density as observed in the previous generation and (ii) a Gaussian current with a power spectrum proportional to that observed in the previous generation. For input parameters that correspond to balanced input in the network, both the renewal and the Gaussian iteration procedure converge quickly and yield comparable results for the self-consistent spike-train power spectrum. We compare our results to large-scale simulations of a random sparsely connected network of leaky integrate-and-fire neurons (Brunel, 2000) and show that in the asynchronous regime close to a state of balanced synaptic input from the network, our iterative schemes provide an excellent approximations to the autocorrelation of spike trains in the recurrent network.

## 1. Introduction

Neurons in different parts of the nervous system respond to repeated presentation of the same stimulus with considerable trial-to-trial variability (van Steveninck et al., 1997). There are several true noise sources contributing to this variability: fluctuations of stochastic ion channels (Schneidman et al., 1998; White et al., 2000), unreliability of synaptic connections such as transmission failure and spontaneous release (Branco and Staras, 2009), and Johnson noise (Manwani and Koch, 1999). These are true noise sources in the sense that they result from the finite number of stochastic elements in the system, be it ionic channels, transmitter molecules, or charge carriers. In cases where synaptic input is absent, e.g., in the neural periphery, the statistics of spontaneous spiking is mainly shaped by channel noise (see e.g., Fisch et al., 2012 for an example); Johnson noise seems to be negligible in many cases (Manwani and Koch, 1999).

Besides these true noise sources there is another source of variability that is most likely dominating for neurons embedded in a network: the quasi-random input from other cells (Destexhe et al., 2003). In contrast to the aforementioned true noise sources, it is not *per se* clear what the input from other cells constitutes: mainly irregular uncontrollable fluctuations (London et al., 2010) or signals, possibly in a highly processed way (Stein et al., 2005; Droste et al., 2013; Masquelier, 2013). No matter how these fluctuations are interpreted, however, it appears reasonable that one may describe them in a stochastic framework and that the statistics of this irregular input is relevant for information transmission and processing in neural networks.

On the theoretical side, unstructured networks with random connections have been studied for a long time (Abbott and van Vreeswijk, 1993; Gerstner, 1995; van Vreeswijk and Sompolinsky, 1996; Brunel and Hakim, 1999; Fusi and Mattia, 1999; Brunel, 2000; Latham et al., 2000; Hansel and Mato, 2003; Leibold, 2004; Burkitt, 2006; Câteau and Reyes, 2006; Brunel and Hakim, 2008; Hennequin et al., 2012; Grytskyy et al., 2013; Ostojic, 2014). Besides various types of oscillatory and/or synchronous behavior, these networks typically also show asynchronous irregular firing if both excitatory and inhibitory connections are included, and excitation and inhibition in the network balance each other (van Vreeswijk and Sompolinsky, 1996). The irregular firing patterns observed in this asynchronous state resemble those of some cortical neurons seen in experiments (Bair et al., 1994; Compte et al., 2003).

An advanced mathematical treatment of stochastic activity in unstructured networks is based on the Fokker-Planck equation. The main assumption for this approach is that the input to the single cell can be described by white Gaussian noise, the mean and noise intensity of which is self-consistently determined by the firing rates of the neurons in the network. Put differently, the mean value and fluctuation intensity of the input spike trains reflect the statistics of the output and the connectivity of the network, where the latter is determined by the number and nature of the connections as well as the synaptic strengths. The Fokker-Planck approach has allowed for many insights into the transition between various states according to the emergence of oscillations and the degree of synchrony (Brunel and Hakim, 1999; Brunel, 2000; Brunel and Hansel, 2006). It has been recently extended to the study of strongly heterogeneous network states (Ostojic, 2014).

As mentioned above, a necessary approximation when using the Fokker-Planck approach in its simplest version is the assumption that the stimulus seen by a single neuron in the network is white Gaussian noise. This is usually justified by the diffusion approximation for a superposition of weakly correlated Poissonian spike trains. However, the spike trains generated by single neurons in the recurrent network are rarely Poissonian, i.e., they display a temporal correlation similar to the experimentally observed ones (Bair et al., 1994) or, equivalently, a non-flat spike-train power spectrum. It is simple to show that the superposition of independent non-Poissonian spike trains inherits the correlations seen in the single spike train (Lindner, 2006). Furthermore, the non-Poissonian nature of spike trains can have severe consequences, e.g., for the output spike-train statistics (Ly and Tranchina, 2009; Schwalger et al., submitted) or for the propagation of signals in feedforward networks (Câteau and Reyes, 2006).

One way to deal with temporal correlations in the input is to extend the phase space of the Fokker-Planck equation by additional variables that can account for colored noise in the input. This has been done by Câteau and Reyes (2006) for the case of green noise (high-pass filtered noise) that arises by a presynaptic refractory period and it can be generalized and utilized to relate output spike-train statistics to temporal input statistics for a simple perfect integrate-and-fire neuron model (Schwalger et al., submitted). Another approach assumes a high degree of intrinsic or external uncorrelated noise that allows for a continuous rate-equation-like description of the activity in the neural network (see e.g., studies by Doiron et al., 2004; Lindner et al., 2005b; Pernice et al., 2011; Trousdale et al., 2012 for networks of integrate-and-fire neurons and the recent review by Grytskyy et al., 2013 for other network types). In this essentially linear description, a connection between input and output correlation matrix is easily derived but the main assumption of the approach, the linearization ansatz, is difficult to justify in general.

**Figure 1:**
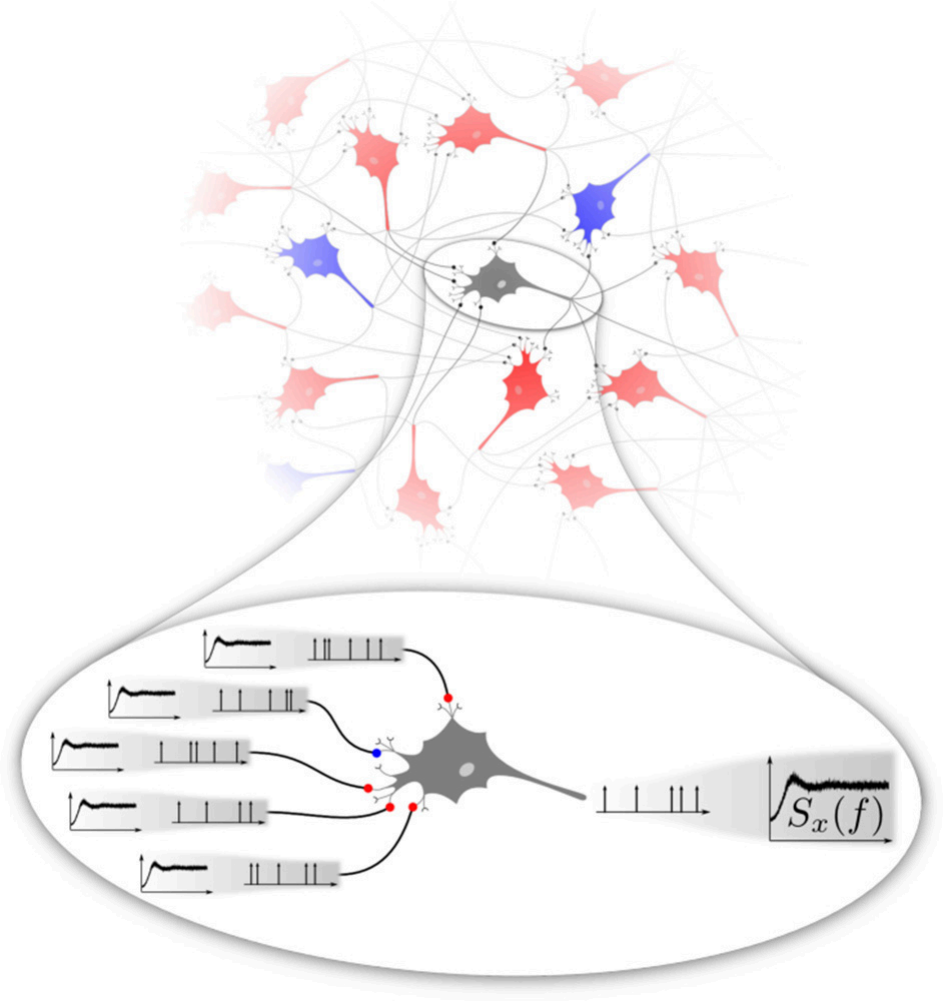
**Basic problem addressed in this paper**. Excitatory (red) and inhibitory (blue) neurons interacting in a recurrent network (top) fire spike trains with a temporal correlation that can be characterized by the spike-train power spectrum. We focus on a homogeneous network, in the sense that excitatory and inhibitory neurons share the same firing rate and power spectrum. At the single-cell level (magnification at the bottom), a neuron is driven by a superposition of spike trains, the power spectra of which should be equal to the power spectrum of the neuron itself. This poses a self-consistency problem that we attempt to solve numerically in this paper in different approximations.

If the stochasticity of neural firing arises mainly from the network input, the following self-consistency problem emerges (cf. Figure 1). For any neuron randomly picked from a homogeneous recurrent network, the second-order statistics of the input spike trains can be characterized by their input power spectra shown in the magnification Figure 1 on the left. These spectra are in general not as flat as that of a (temporally uncorrelated) Poisson process. They should match the statistics of the neuron itself, in Figure 1 represented again by the same non-flat power spectrum shown on the right. There are obvious generalizations possible if we think of different types of neural subpopulations of neurons sharing a common spike-train statistics (e.g., firing rates and power spectra), which all must be consistent with each other depending on the topology of the network. Even in the simple homogeneous version of the problem, the question has some interest on its own: What is the temporal correlation of a shot noise that would evoke a neural output with the same correlation statistics? Mathematically, it is not even clear whether such a solution exist and if so whether it is unique.

Lerchner et al. (2006) suggested a simple numerical procedure to determine a self-consistent autocorrelation statistics of spike trains in a sparse network using a Gaussian approximation. They focused on the case of network input that is balanced between excitation and inhibition and studied the dependence of the Fano factor on synaptic strength.

In this paper, we use similar self-consistent numerical procedures to determine the temporal correlations of single neuron activity in a sparse network of excitatory and inhibitory neurons in the asynchronous state. We employ exclusively the leaky integrate-and-fire (LIF) model (Lapicque, 1907; Gerstner and Kistler, 2002), that has been a standard choice in many studies of recurrent networks.

We use an iterative numerical scheme to determine the self-consistent second-order statistics of spike trains in a recurrent neural network. In the *n*th step (henceforth referred to as the *n*th generation) of this procedure, we stimulate an LIF neuron in repeated trials with noisy input, the statistics of which is determined from the spike statistics of the previous generation. In order to generate the input, we employ two approximations. In one version, we use the ISI density of the LIF neuron to generate driving renewal spike trains for the next generation (renewal approximation). In an alternative version (equivalent to the original idea by Lerchner et al., 2006), we generate a Gaussian process that has the same power spectrum as the LIF spike train to generate the input for the next generation (Gaussian approximation).

For a parameter regime of balanced excitatory and inhibitory input from the previous generation, the spike-train power spectrum of the LIF neuron converges quickly over a small number of generations to a stationary spectrum. If the inhibitory component is too strong, however, our iterative scheme does not converge but displays strong oscillations in the firing rate as a function of the number of generations. As we will show, this instability can be understood already within the framework of the diffusion approximation.

We furthermore present results of extensive simulations for a sparse recurrent homogeneous network of excitatory and inhibitory LIF neurons, using parameters similar to the classical study by Brunel (2000). In the regime where our approximation scheme converges, we compare the power spectra to results from the renewal and Gaussian approximations. We find close agreement of power spectra for parameters of the Brunel setup for which the activity of neurons is asynchronous and the total input coming from the network is almost balanced. We conclude by discussing the implications of our results for a more faithful description of neural noise emerging in recurrent networks.

## 2. Models and methods

### 2.1. Model of the single neuron and spike-train statistics

We consider a leaky integrate-and-fire model receiving an input current *I*(*t*) (here multiplied by the membrane resistance *R*) that obeys the following dynamics

(1) $$\tau \dot{v}=-v+RI(t).$$

where the membrane time constant is chosen τ = 20 ms throughout this paper. Whenever the voltage reaches the threshold of *v_T_* = 20 mV, a spike time *t_i_* is registered, and after an absolute refractory period of τ_ref_ = 2 ms the voltage is reset to a value *v_R_*, for which we use two different values (0 and 10 mV, see results). The current *I(t)* differs according to whether we consider a recurrent network or our self-consistent approximation schemes. In all cases considered, we numerically integrate Equation 1 with a simple Euler scheme using a time step of Δ*t* = 0.1 ms. Please note that in all models studied in this paper, there is no Gaussian white noise, which would require a smaller time step.

The spike times defined by threshold crossings can be used to determine the statistics of the interspike interval (ISI) *I_i_* = *t_i_* − *t*_*i* − 1_. The statistics inspected in this paper are (i) the mean interval 〈*I_i_*〉 (〈·〉 indicates an ensemble average), which is related to the firing rate by ν = 1/〈*I_i_*〉; (ii) the coefficient of variation (CV)

(2) $$C_{V}=\frac{\sqrt{\langle {\left(I_{i}-\langle I_{i}\rangle \right)}^{2}\rangle }}{\langle I_{i}\rangle },$$

and (iii) the serial correlation coefficient among intervals that are lagged by an integer *k*:

(3) $$\rho_{k}=\frac{\langle \left(I_{i}-\langle I_{i}\rangle \right)\left(I_{i\;+\;k}-\langle I_{i\;+\;k}\rangle \right)\rangle }{\langle {\left(I_{i}-\langle I_{i}\rangle \right)}^{2}\rangle }.$$

The neural spike train is represented by a sum of delta functions at the spike times

(4) $$x(t)=\sum_{i}\delta (t-t_{i}).$$

The spike-train power spectrum is computed from the Fourier transform of the spike train by

(5) $$S(f)=\frac{\langle \tilde{x}{\tilde{x}}^{*}\rangle }{T},$$

where the Fourier transform for the time window is defined by

(6) $$\tilde{x}(f)=\int_{0}^{T}dte^{2\pi ift}x(t).$$

For the recurrent network, we assume that all neurons are statistically equivalent and that we can both average over realizations of initial conditions in the membrane voltage of the single cell *and* over different neurons when computing power spectra according to Equation 5 as well as all other spike-train measures employed in this work.

### 2.2. Recurrent-network model

We consider a connected random network of *N*_E_ excitatory and *N*_I_ = γ *N*_E_ inhibitory LIF neurons as studied by Brunel (2000) in his model A. As the only topological constraint, excitatory and inhibitory neurons are assigned the same number *C*_E_ and *C*_I_ = γ *C*_E_ of presynaptic excitatory and inhibitory neurons, respectively. Both neuron types follow the same single-cell dynamics; all parameter values of the LIF model are identical. This setup can still be regarded as homogeneous in the sense that in a large and sparse network power spectra of excitatory and inhibitory neurons should coincide, as verified numerically for all used parameter values. Spectra in recurrent networks presented here are averaged over 10^3^ randomly picked neurons.

In the network simulations, the voltage variables *v*_ℓ_(*t*) with ℓ = 1, 2, …, *N*_E_(1 + γ) all obey the same dynamics Equation 1 and fire-and-reset rule as explained above. The input current (*I*_ℓ_(*t*) = *I*_ℓ,loc_(*t*) + *I*_ℓ,ext_(*t*) to the ℓth neuron in Equation 1 consists of a local part

(7) $$\begin{array}{l} RI_{\ell ,\text{loc}}(t)=\tau \sum_{j\;=\;1}^{C_{\text{E}}}J\sum_{i}\delta (t-t_{\ell ,i,j}-D)\\ \;-\tau \sum_{j\;=\;1}^{C_{\text{I}}}gJ\sum_{i}\delta (t-t_{\ell ,i,j}-D), \end{array}$$

comprising the input current from *C*_E_ presynaptic excitatory and *C*_I_ inhibitory neurons in the network. Here *g* is the relative strength of inhibitory amplitudes. The time instance *t*_ℓ,*i,j*_ denotes the *i*th spiking time of the *j*th presynaptic neuron of the ℓth postsynaptic cell. The transmission delay is denoted by *D*. For the external input, we either consider a Poissonian background noise

(8) $$RI_{\ell ,\text{ext}}(t)=\tau \sum_{j\;=\;1}^{C_{\text{E}}}J\sum_{i}\delta (t-t_{\ell ,i,j}),$$

from an external population of excitatory neurons (to be consistent with Brunel, 2000) or a constant input current equal to the mean of the Poisson input:

(9) $$RI_{\text{ext}}(t)=C_{\text{E}}J\nu_{\text{ext}}\tau .$$

We will use the constant external input current Equation 9 if not stated otherwise, in order to focus on noise (stochasticity) that is generated solely by the internal dynamics of the network itself.

For the standard network parameters, we follow (Brunel, 2000) by using γ = 0.25, *C*_E_ = 10^3^, *D* = 1.5 ms, *J* = 0.1 mV, but choose a larger network size of *N*_E_ = 10^5^ (Brunel, 2000 used *N*_E_ = 10^4^). Note that our value of γ implies that the input from the recurrent network is balanced if *g* = 4. Furthermore, we choose the constant external input such that *RI*_ext_(*t*) = 30 mV, which corresponds in Brunel (2000) to ν_ext_/ν_thr_ = 1.5, (ν_thr_ is the frequency of the external Poisson input needed to set the mean membrane potential to *v_T_* in the absence of local synaptic input). With this choice of parameters, the network is in the asynchronous firing regime for the range of values of *g* considered in our study (*g* ∈ [3.5, 5]).

### 2.3. Self-consistent determination of spectral statistics

The numerical procedure to determine the self-consistent spectral statistics uses essentially only a single model neuron in a number of repeated simulations. First, the neuron is stimulated by a combination of constant input and a Poisson process with given rate. A sufficient number of trials is carried out to reliably determine the output statistics of the neuron. This constitutes the output statistics of the *first generation* (the Poissonian drive can be regarded as the zeroth generation). In the next step we generate surrogate input to the neuron of the *second generation* according to one of the two approximations explained below. Again this is repeated for as many trials as required to obtain a reliable output statistics. The latter is used once more to generate surrogate data for the *third generation* and the whole procedure is repeated until the spike-train statistics converges, i.e., until the power spectrum of the *n*th generation does not differ significantly anymore from that of the (*n* − 1)th generation.

Our procedure is completely equivalent to simulating a feedforward network, in which layers correspond to the generations. There are two peculiarities compared to the usual setup of feedforward networks. First, in the way we approximate the input, all spatial correlations within a layer are neglected. Secondly, the number of layers is solely determined by the convergence of the spectra.

Because it is difficult to generate surrogate data with exactly the same statistics as the output of the previous generation, we employ two different approximations for the input, which are explained in the following subsections.

#### 2.3.1. Gaussian approximation for the input of the next generation

As an extension of the diffusion approximation the local spike-train input is approximated by a Gaussian noise η(*t*) ≈ *RI*_loc_(*t*), that is, however, not uncorrelated (white) as it would be in the diffusion approximation. The mean value is given by the constant current 〈η〉 = *C*_E_*J*(1 − *g*γ)ντ, which represents the average of the overall local input current. The power spectrum of the Gaussian noise equals the one of the summed spike trains of all presynaptic neurons. With the assumption of independent neurons this yields *S*_η_(*f*) = (*C*_E_*J*^2^ + *C*_I_*g*^2^*J*^2^)τ^2^*S_x_*(*f*), where *S_x_*(*f*) is the spike-train power spectrum of the previous generation.

The approximation only requires to measure the power spectrum in each generation. Surrogate Gaussian input for the next generation that has this power spectrum can then be generated with standard algorithms (Billah and Shinozuka, 1990). Briefly, to generate a Gaussian time series η(*t_j_*) of *N* steps of size Δ*t* with a prescribed power spectrum *S*_η_(*f*), we draw in Fourier space in each frequency bin two independent Gaussian random numbers $\tilde{\eta }$_*r*_(*f_k_*), $\tilde{\eta }$_*i*_(*f_k_*) with

(10) $$\langle {\tilde{\eta }}_{m}(f_{k})\rangle =0,\;\langle {\tilde{\eta }}_{m}(f_{k}){\tilde{\eta }}_{n}(f_{\ell })\rangle =\frac{\delta_{m,n}\delta_{k,\ell }}{2\Delta f}S_{\eta }(f_{k}),\;m,n\in \{r,i\}\;\;\;\;\;$$

where *f_k_* and Δ*f* = (*N* Δ*t*)^−1^ are center frequency and width of the *k*-th bin, respectively. By construction, the complex-valued sequence $\tilde{\eta }$(*f_k_*) = $\tilde{\eta }$_*r*_(*f_k_*)+ *i* $\tilde{\eta }$_*i*_(*f_k_*) is uncorrelated between frequency bins and has a variance proportional to the desired power spectrum. Transformation into the time domain (e.g., by fast Fourier transform) then yields the desired time series. Note that the Gaussian approximation assumes a high overall firing rate and a small synaptic efficacy (weight) and is expected to fail if one or only a few input spikes can already elicit an output spike.

The iterative procedure put forward by Lerchner et al. (2006) is similar in nature, drawing surrogate input statistics straight from the previous generation's autocorrelation function. As—unlike there—we consider uniform (i) firing thresholds and (ii) numbers of presynaptic excitary/inhibitory neurons, our LIF dynamics do not yield firing-rate heterogeneities in the network that would need to be accounted for. This in turn considerably simplifies the procedure and speeds up its numerical implementation.

#### 2.3.2. Renewal approximation for the input of the next generation

For this approximation, we also measure the interspike interval histogram along with the spike-train power spectrum. This ISI histogram can be used to generate surrogate spike-train input for the next generation in form of renewal processes that have by construction the same interspike interval histogram. To this end, for each of the *C*_E_ excitatory and *C*_I_ inhibitory input spike trains, we assume an initial spike at *t* = − *T*_0_ and draw in a large time window [−*T*_0_, *T*] a sufficient number of interspike intervals *I_i_* such that ∑*I_i_* > *T* + *T*_0_. Partial sums of one, two, three etc. intervals then yield the first, second, etc spike time of the respective input renewal spike train. Although the intervals of different input spike trains are independent, all *C*_E_ + *C*_I_ renewal processes are initially synchronized by the common initial spike at *t* = −*T*_0_. To achieve a stationary asynchronous ensemble of renewal spike trains (Cox, 1962), we use only the spike trains in the subinterval [0, *T*]. The necessary equilibration period *T*_0_ can be estimated as *T*_0_ ≈ (ν*C*^2^_*V*_)^−1^ (for *C_V_* < 1 as is the typical case in this paper).

As a smart alternative, we may start at *t* = 0 and use as the first spike time *t*_1_ a sample of the so-called forward recurrence (FR) time, the probability density of which can be computed from the ISI density ρ(*t*) as follows (Cox, 1962)

(11) $$\rho_{\text{FR}}(t_{1})=\nu \int_{t_{1}}^{\infty }dt^{\prime }\rho (t^{\prime }).$$

Thus, if we generate the first spike time *t*_1_ from ρ_FR_(*t*_1_) and all the following *t_i_* with *i* = 2, 3, … from drawing intervals according to ρ(*t_i_* − *t*_*i* − 1_), we will also generate an equilibrium renewal spike train, avoiding the simulation period [−*T*_0_, 0] in the first method. We tested that both methods to generate an equilibrium renewal point process yield similar numerical results in our procedure.

Superposition of the *C*_E_ excitatory renewal spike trains with amplitude *J* and *C*_I_ spike trains with amplitude −*gJ* in the time window of [0, *T*] are used to stimulate the LIF model in the *n*th generation. Note that the superposition of the renewal spike trains is in general not a renewal process (Lindner, 2006) and thus there is no simple way to generate surrogate data for the superposition of the renewal spike trains directly instead of generating the single processes and summing them up. Although for special renewal processes (e.g., Gamma processes), efficient algorithms for the generation of such sums exists (Deger et al., 2012), our problem does not allow to specify the nature of the point process in advance. Hence, in particular for large *C*_E_, *C*_I_, the generation of renewal input becomes numerically inefficient.

We expect that the renewal approximation will work well if ISI correlations in the output spike train can be neglected. In contrast to the Gaussian approximation explained above there are no limitations regarding the spike amplitude and input rates. However, it is important to keep in mind that the renewal approximation cannot exactly yield what we are aiming at: a self-consistent second-order statistics because the generation of the surrogate data for the input is based on the ISI statistics and not on the second-order spike-train statistics. Only if also the output spike train is a renewal process, there is a unique relationship between power spectrum and ISI probability density (Stratonovich, 1967):

(12) $$S(f)=\nu \frac{1-|\tilde{\rho }(f){|}^{2}}{|1-\tilde{\rho }(f){|}^{2}},$$

where $\tilde{\rho }$(*f*) is the Fourier transform of the ISI density ρ(*I*). By construction, Equation 12 yields the power spectrum for each of the surrogate input spike trains and is also proportional to the total sum of all independent input spike trains (Lindner, 2006). However, the power spectrum of the output spike train (sharing the same ISI density ρ(*I*)) does not obey Equation 12 unless all (linear but also non-linear) correlations among ISIs can be neglected. Briefly, output spectrum equals input spectrum only if the output spike train is also a renewal process. For finite ISI correlations, we can expect a discrepancy between the power spectrum of the surrogate input (superposition of renewal processes) and the power spectrum of the output spike train (which is in general non-renewal), even if our scheme has converged to a stationary output spike train. In contrast to the Gaussian approximation, it is more difficult to estimate when the renewal approximation will fail because it does depend on a property of the output (interval correlations) and not on the input (e.g., the size of amplitudes as for the Gaussian approximation).

#### 2.3.3. Convergence and uniqueness of the algorithms

In general we consider Poisson spike trains as the input for the first generation. This input has only one parameter, the firing rate of the Poisson process. To see the difference between what we would obtain in the diffusion approximation, we use the firing rate determined in the network simulations below. The firing statistics of the first generation is then close to what we would expect to see in the diffusion approximation. Conveniently, differences between the converged power spectrum and the power spectrum of the first generation correspond to differences between the actual and the approximated output spectra in a theory based on the Poisson assumption.

We have tested in several cases that as long the procedure is stable (see below), the initial statistics does not matter and converged spectra are the same whether we start with asynchronous periodic input (*C*_E_ + *C*_I_ completely periodic spike trains with randomized initial spike times) or with Poisson input with a firing rate that differs significantly from the asymptotic value. So as far as numerical evidence in a limited parameter regime can tell, the procedure (if stable) converges to a unique spike-train power spectrum power spectrum.

One simple condition for the convergence of power spectra is that an even more essential statistics, the firing rate, converges. As our scheme can be regarded as a map, for which the firing statistics of the (*n* − 1)th generation determines that of the *n*th generation, we have to require that this map possesses a stable fixed point. Because the diffusion approximation captures this first-order statistics of the spike train fairly well (Brunel, 2000), we can employ the rate formula to estimate the map between the input rate (from the (*n* − 1)th generation) to the output rate (that of the *n*th generation by the well-known formula for the rate of a white-noise driven LIF neuron (Brunel, 2000)

(13) $$\nu_{\text{out}}={\left(\tau_{\text{ref}}+\tau \sqrt{\pi }\int_{\frac{\mu (\nu_{\text{in}})\;-\;v_{T}}{\sqrt{2D(\nu_{\text{in}})}}}^{\frac{\mu (\nu_{\text{in}})\;-\;v_{R}}{\sqrt{2D(\nu_{\text{in}})}}}dze^{\;z^{2}}\text{erfc(z)}\right)}^{-1}\text{​}.$$

Here the constant input

(14) $$\mu (\nu_{\text{in}})=\langle RI(t)\rangle =C_{\text{E}}J\tau [\nu_{\text{ext}}+(1-\gamma g)\nu_{\text{in}}]$$

and the intensity of the white Gaussian noise

(15) $$D(\nu_{\text{in}})=\frac{C_{\text{E}}J^{2}\tau (1+\gamma g^{2})\nu_{\text{in}}}{2}$$

both depend on the input rate ν_in_ (note that we assumed just a constant external input, which does not contribute to the noise). A stable fixed point of the map should be characterized by the equality of input and output rates ν_out_(ν_in_) = ν_in_, which become apparent as intersection points of the graph ν_out_(ν_in_) with the diagonal. Additionally, we have to require at this intersection point a slope |*d*ν_out_/*d*ν_in_| < 1 to ensure that small perturbations in the firing rate decay.

## 3. Results

### 3.1. Self-consistent spectrum using two different iterative schemes

**Figure 2:**
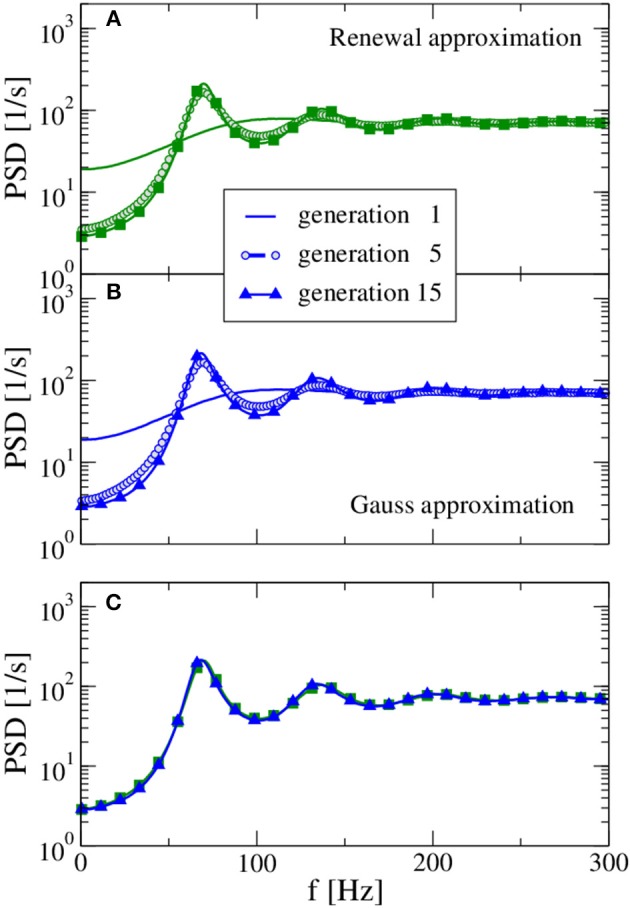
**Power spectra resulting from the self-consistent procedure**. For balanced input from the previous generation (*g* = 4) and a large presynaptic environment (*C*_E_ = 10^3^, *C*_I_ = 250) both the renewal approximation **(A)** and the Gaussian approximation **(B)** have converged to unique stationary spectra, which are compared in **(C)**. In the first generation, the neuron is stimulated by a constant input 〈*RI(t)*〉 = 30 mV and *C*_E_ + *C*_I_ Poissonian spike trains of rate ν_in_ = 71 Hz [solution for the self-consistent firing rate Equation (13)] with amplitude *J* = 0.1 mV (excitatory synapses) and −*gJ* = −0.4 mV (inhibitory synapses). Note the rapid convergence of spectra for both approximations: the spectrum of the fifth generation differs only slightly from the result for the 15th generation.

We begin with an example for which our procedure leads to a stable stationary output spike-train statistics (in terms of firing rate and spike-train power spectrum) and where both approximations yield very similar spectra. In Figure 2 we show the power spectra of the selected generations using the renewal approximation in A, the Gaussian approximation in B, and compare the asymptotic spectra of both models in panel C. The number of presynaptic neurons corresponds here to the standard values of *C*_E_ = 1000, *C*_I_ = 250 used by Brunel (2000), while the strength of inhibition *g* = 4 is chosen such that the network input is balanced.

In the first generation the neuron is stimulated by a Poisson spike train with a self-consistent firing rate^1^ according to the stable fixed point of Equation 13. The power spectrum of the first generation (solid lines in Figure 2) gives us what we would expect in the Poisson approximation of neural background activity: a spectrum with reduced power at low frequency, indicative of a stochastic process that one may refer to as a “green noise” (Guz and Sviridov, 1998). This spectrum agrees remarkably well with the analytical expression of the power spectrum of a white-noise driven LIF (Lindner et al., 2002) with the effective base current and noise intensity given by Equation (14) and (15), respectively (not shown).

On the contrary, the self-consistent power spectrum of the 15th generation is a narrow-band noise with strong peaks around frequencies equal to the firing rate or multiples of it. In the self-consistent picture, the neuron of the 15th generation is not driven by a spectrally flat noise but by a narrow-band noise with power around its firing rate that apparently leads to a much more regular spike train than an uncorrelated noise (the Poisson spike train) does.

**Figure 3:**
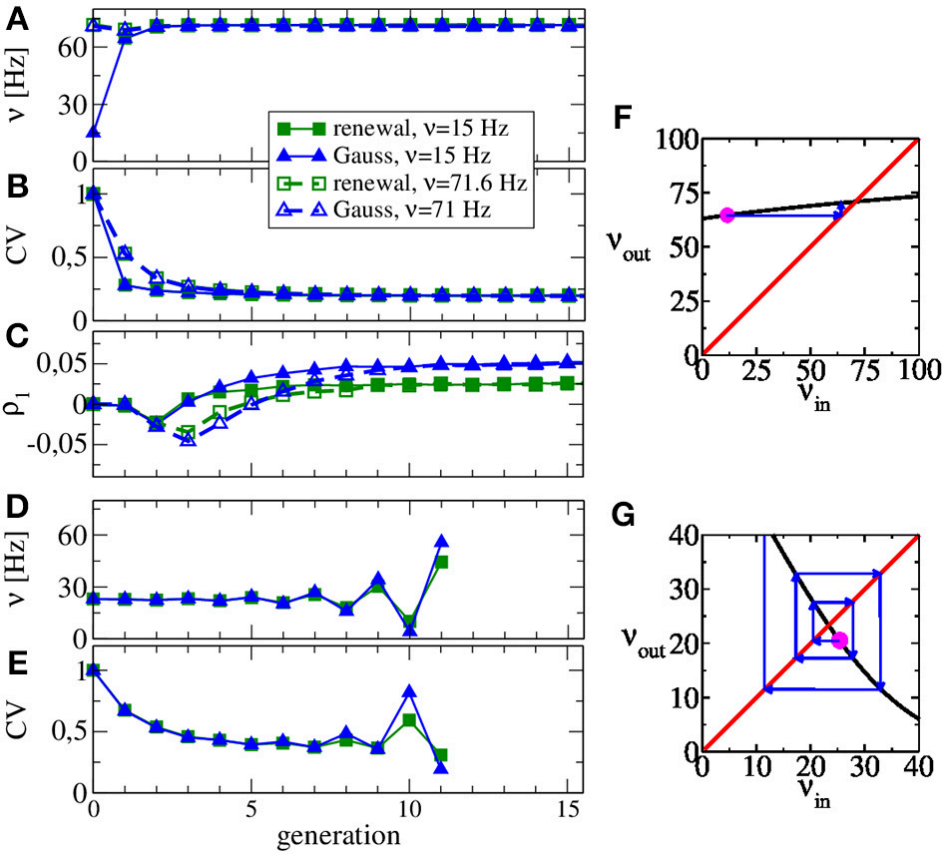
**Evolution of ISI statistics over generations in stable **(A,B,C,F)** and unstable (D,E,G) regimes**. Starting with Poissonian spike trains in the zeroth generation, the *n*th generation of the LIF neuron (*n* ≥ 1) receives noise input according to the statistics of the previous generation. Parameters as in Figure 2 yield the same stable rate **(A)** and CV **(B)** irrespective of whether the initial Poisson stimulation (zeroth generation) of the first generation (LIF neuron) is 15 or 71 Hz. The first serial correlation coefficient is positive for both procedures but differs in magnitude **(C)**. Increasing the relative strength of inhibition to *g* = 5, our scheme is not stable anymore and both rate **(D)** and CV **(E)** oscillate as functions of the generation. Stability can be discussed in terms of the firing rate Equation 13 shown in **(F,G)** vs. input rate (black line) together with the identity line. In the regime of **(A–C)**, the map from input rate to output rate **(F)** has a stable fixed point and small perturbations from it (magenta point) relax back into the fixed point (blue arrows). In the regime of **(D–E)**, small perturbations are amplified **(G)**, yielding an unstable fixed point.

In Figures 3A,B we show the rate and the CV as functions of the generation, respectively. We use Poisson processes to generate the input to the first generation, once with a firing rate close to the asymptotic one (ν_in_ = 71 Hz), once with a substantially lower rate (ν_in_ = 15 Hz). Apparently, the converged statistics after 15 generations do not depend on the initial value of the rate. While the firing rate does not change much over the generations, the CV drops from a value of 0.5–0.2. Hence the diffusion approximation (equivalent to the statistics of the first generation) leads to a reliable estimate of the self-consistent value of the firing rate but not of the CV. This discrepancy was already evident by looking at the power spectra at low frequency, which is largely determined by the CV according to *S*(0) = ν *C*^2^_*V*_ (true only for a renewal process). Figure 3F illustrates the reason for the rapid convergence of our procedure over the generations in terms of the map for the firing rate Equation 13. The shallow dependence of the firing rate curve ν_out_(ν_in_) around the intersection point with the diagonal shown in Figure 3F implies that any initial perturbation from the fixed point (indicated by the magenta spot) approaches the fixed point monotonically over only a few generations (blue arrows).

Interestingly, although renewal and Gaussian approximations yield similar results for rate, CV, and power spectra, they differ in the stationary value of the first serial correlation coefficient ρ_1_, displayed in Figure 3C. This value is positive for the considered parameter set but in the renewal scheme we obtain only half the value of the correlation coefficient, which is observed when we use the Gaussian approximation and which is also close to the value observed in network simulations.

The map for the firing rate can also be used to understand why our procedure does not work for very strong inhibition. This case is illustrated in Figures 3D,E for *g* = 5, for which we observe oscillations in rate and CV that grow in amplitude over the generations (no instabilities are observed in the recurrent network for these parameters). Here the map for the firing rate still has a fixed point but it is an unstable one, i.e., |*d*ν_out_(ν_in_)/*d*ν_in_| > 1 at the fixed point and small perturbations from the fixed point grow in amplitude (cf. Figure 3G). Interestingly, the stability also depends on the size of the presynaptic environment, even if we fix the mean input from the previous generation because the number of synapses also determine the effective noise level Equation 15. For instance, a smaller presynaptic environment with *g* = 5, *C*_I_ = 100, *C*_I_ = 25 and *J* = 1 mV (leading to the same mean input as our standard parameter), the slope of the firing rate curve is still negative but its absolute value is smaller than one. Hence, here our procedure still yields a self-consistent spike-train power spectrum in this case (not shown).

For the parameter set in Figure 2 both approximations yielded the same power spectrum because their respective assumptions, i.e., small amplitudes *J* for the Gaussian approximation and independence of ISIs for the renewal approximation, were sufficiently closely matched. Below in Section 3.3 we will show two examples, for which the two approximations result in visibly distinct power spectra because one of their respective assumptions is not obeyed.

### 3.2. Spectra in recurrent networks

We would like to compare our results for self-consistent spectra to those measured in a recurrent neural network. Here we use the model by Brunel (2000) (more specifically, Brunel's model A) within the parameter regime of asynchronous activity. Because we want to focus on the sparse limit of the model, in which input correlations can be neglected, we choose as a standard network size *N*_E_(1 + γ) = 1.25 · 10^5^ (exceeding the one used by Brunel, 2000 by a factor of 10). We clarify to what extend spike-train power spectra in the recurrent network depend on the transmission delay, the network size, and whether they are robust with respect to external noise.

In the approximation schemes introduced above, there is no synaptic delay *D*: the statistics of the (*n* − 1)th generation are measured during an ideally very large time interval, and stationary stochastic input with similar statistics is used to stimulate the neuron model in the *n*th generation; introducing here a delay would have no consequences for the spike statistics of the *n*th generation at all. In contrast, in the recurrent network, degree and character of synchrony in firing patterns depend strongly on *D* (Brunel, 2000).

**Figure 4:**
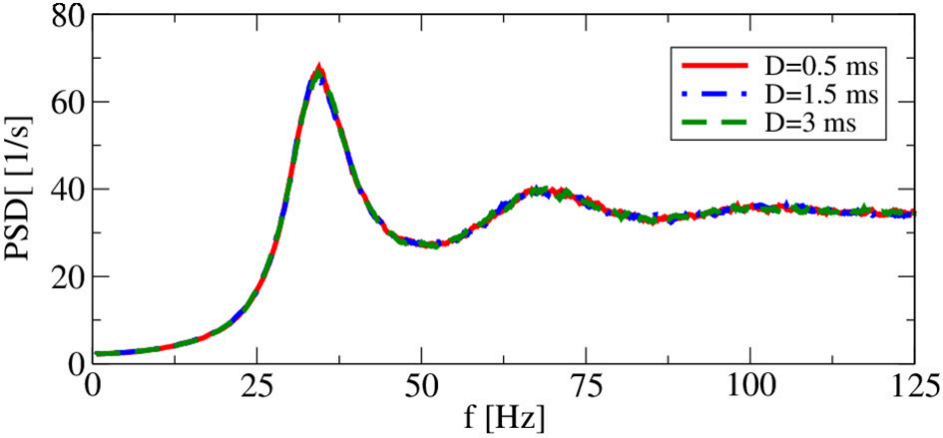
**Delay dependence of power spectra in recurrent networks**. Asynchronous regime. Parameters: (*g* = 4.5), and (*v*_R_ = 10 mV).

It turns out, however, that for a range of synaptic delays in the asynchronous firing regime, single-neuron statistics are independent of the particular choice of *D*. As shown in Figure 4, power spectra for delays that differ by a factor of 6 are very close to each other. Thus, in the iterative procedure, a self-consistent determination of the power spectrum in the asynchronous regime is possible without incorporating a delay *D*.

**Figure 5:**
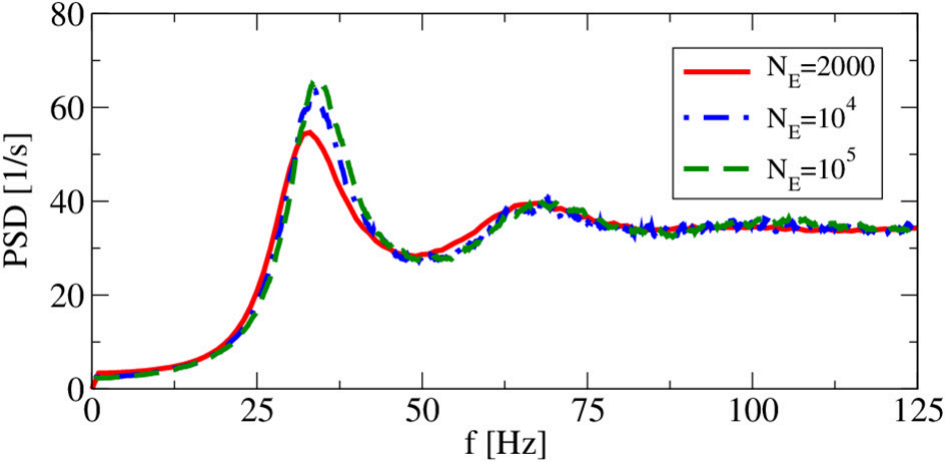
**System-size dependence of power spectra in recurrent networks**. Asynchronous regime for (*g* = 4.5), and (*v*_R_ = 10 mV).

Apart from the synaptic delay, also the network size does not explicitly appear as a model parameter in our iterative procedures. However, implicitly we have assumed in both approximations that cross-correlations can be neglected, which in the recurrent network can be achieved (if at all) by a large network size. Hence, size is a concern and we have to check, how spike-train power spectra change as we change the system size at fixed number of input neurons. This is illustrated in Figure 5, where the smallest system size *N*_E_ = 2000 implies with *C*_E_ + *C*_I_ = 1250 a non-negligible overlap of input neurons for any two neurons and thus significant cross-correlations among the neurons. However, for network sizes *N*_E_ = 10^4^ (as used by Brunel, 2000) and *N*_E_ = 10^5^, the spike train power spectra look very similar, justifying the choice of *N*_E,I_ used in the following.

**Figure 6:**
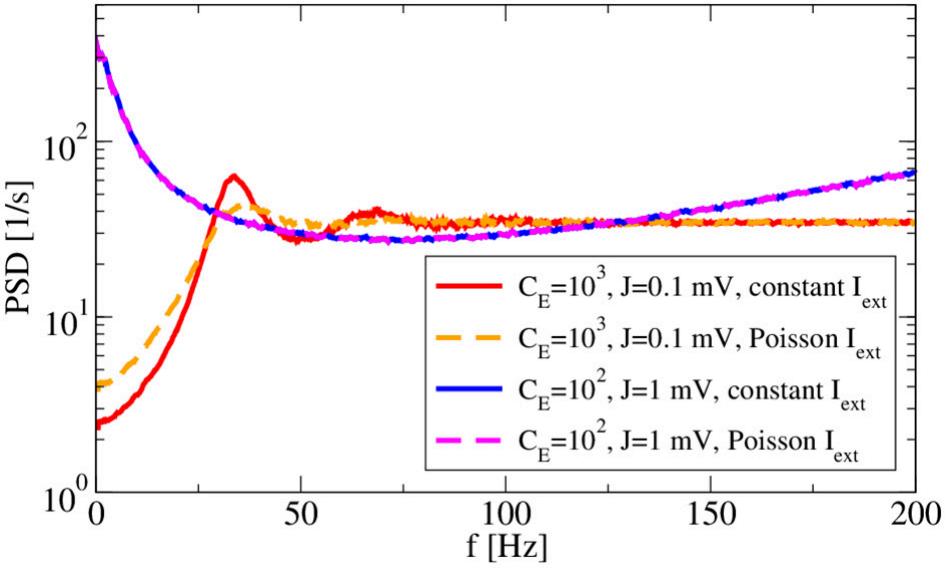
**Synaptic-amplitude dependence of power spectra and difference between external constant and shot-noise input**. Asynchronous regime for (*g* = 4.5) and (*v*_R_ = 10 mV).

Two more features of the system are inspected in Figure 6: the robustness to external input and the dependence of spectra on the spike amplitude *J*. With respect to the latter, we use, besides our standard choice *J* = 0.1 mV with *C*_E_ = 1000, *C*_I_ = 250, also a larger amplitude of *J* = 1 mV with a reduced number of presynaptic neurons (*C*_E_ = 100, *C*_I_ = 25) such that the mean input from the network remains the same. Note that our change of parameters is different to that considered by Ostojic (2014), because we reduce the number of synapses when increasing the amplitude, avoiding in this way strong fluctuations in the population rate as seen by Ostojic (2014). Increasing the amplitude in our setting has the main effect of increasing the noisy input for the single neuron, which leads in our setup to a bursting behavior that becomes apparent by increased power at low frequency. Replacing the constant input current μ with an external Poissonian stimulus of the same mean generally does not alter the firing regime (Brunel, 2000) because this noise is only small compared to that coming from the recurrent network. In fact, for *J* = 1 mV the spectra with external Poisson spikes (dashed magenta line in Figure 6) and with a constant input of the same mean (blue line) do not differ at all. The effect of such an external noise is more visible for our standard choice: peaks in the power spectrum (dashed orange line) become wider and the power at low frequency is increased compared to the spectrum with a constant external input current (red line). These are expected effects of external white noise on the power spectrum of a spike generator in the mean-driven regime (see e.g., Lindner et al., 2002).

After we have seen that power spectra in the recurrent network neither depend on the specific value of delays (as long as the existence of the asynchronous regime is ensured) nor on network size (as long as it is large enough), and that they do not change drastically with additional external noise, we turn now to the comparison of network spectra with the spectra from the self-consistent procedure.

### 3.3. Comparison of spectra in recurrent networks and the self-consistent solution

Besides the comparison to the results of our iterative scheme, we use this section also to additionally inspect the variation of another parameter, the reset potential *v_R_*. So far we have chosen *V_R_* = 10 mV in accordance with (Brunel, 2000), corresponding to a voltage that is reset between the resting potential and the threshold. This is a reasonable choice for some cortical cells (Koch, 1999), but a reset closer to the equilibrium potential may be also appropriate for others. Hence, it is of interest how power spectra and also how our approximation schemes for them may depend on the choice of *v_R_*. We will thus use in all plots of this subsection *v_R_* = 0 mV as an alternative setup. Based on previous work (Vilela and Lindner, 2009) we can expect that with this value of the reset, we will observe a lower firing rate and also a lower CV than for *v_R_* = 10 mV.

**Figure 7:**
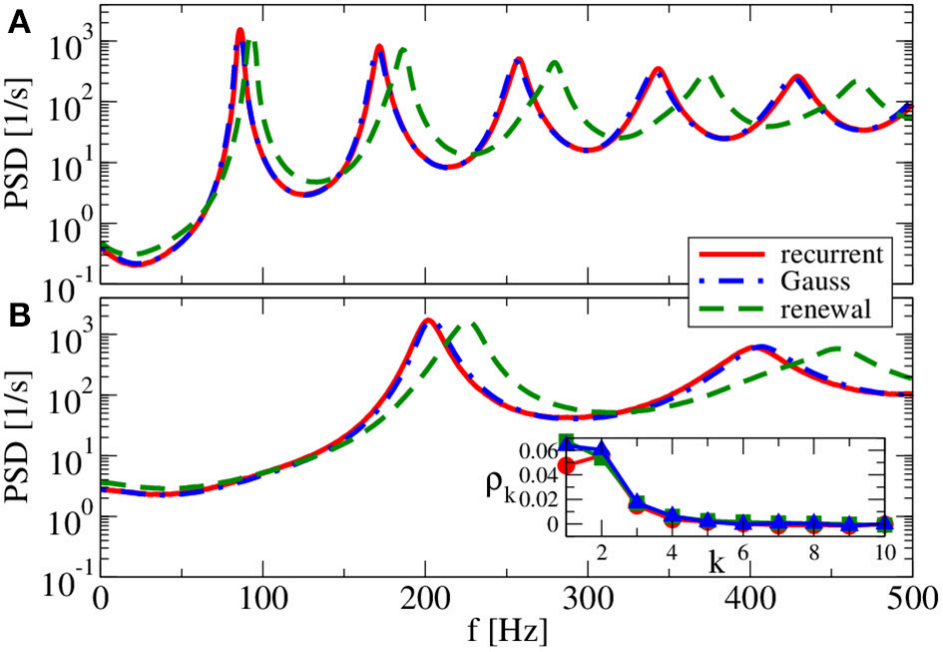
**Power spectra for dominating network excitation**. Results of recurrent network simulations and the two approximations from our iterative schemes for *g* = 3.5 (excitatory local synaptic input). **(A)** *v*_R_ = 0 mV (firing rate ν = 86.1 Hz, *C_V_* = 0.045); **(B)** *v*_R_ = 10 mV (firing rate ν = 202.5 Hz, *C_V_* = 0.10). Inset shows serial correlation coefficients for **(B)**.

Our main parameter to vary in the following is the relative strength of inhibition *g*. We start with a value of *g* = 3.5 (see Figure 7), which is close to the border of synchronization (Brunel, 2000). For *g* = 3.5, the spectra reveal strong peaks, i.e., although neurons still fire asynchronously, their spike trains are rather regular. The Gaussian approximation leads in our self-consistent procedure to a spectrum that captures the spike-train power spectrum of a neuron in the recurrent network very well. This holds true for both a reset value of *v_R_* = 0 mV (panel A) and for *v_R_* = 10 mV (panel B). As anticipated above the firing rate is higher for *v_R_* = 10 mV and, consequently, also spectral peaks are located at higher frequencies.

In contrast to the Gaussian approximation, for *g* = 3.5 the renewal approximation used in our iterative scheme does not yield a power spectrum that closely matches the spectrum in the recurrent network. Peaks appear here at a somewhat higher frequency, and the neuron also fires at a somewhat higher rate. This discrepancy can be traced back to a non-renewal behavior indicated by the positive ISI correlations at lag one and two (cf. inset in Figure 7B for *v_R_* = 10 mV).

**Figure 8:**
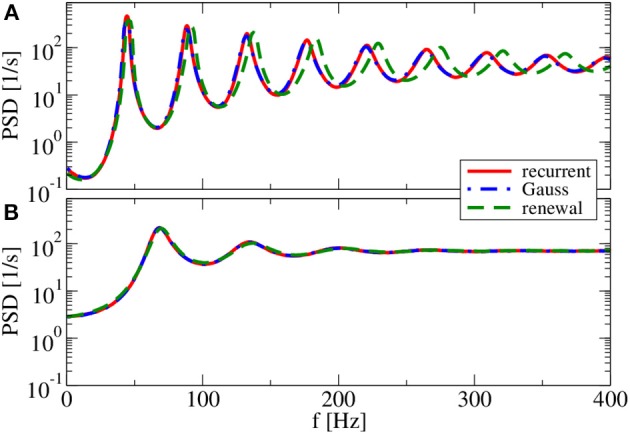
**Power spectra for balanced network input**. Results of recurrent network simulations and the two approximations from our iterative schemes for (*g* = 4) (balanced local synaptic input). **(A)** (*v*_R_ = 0 mV) [firing rate ν = 44.5 Hz, *C_V_* = 0.05] and **(B)** (*v*_R_ = 10 mV) (firing rate ν = 70.4 Hz, *C_V_* = 0.19).

As we increase the relative strength of inhibition to *g* = 4, both approximations agree well with the spectrum measured in the recurrent network if we use the reset voltage of *v_R_* = 10 mV (cf. Figure 8B). This is not totally unexpected because for these parameters we found already an agreement of both approximations in Figure 2C. Because of the complementary assumptions made in the two approximations, an agreement of their self-consistent spectra is a strong hint that they both should work—Figure 2C can be taken as a confirmation of this.

Interestingly, if we choose the reset value at *v_R_* = 0 mV (cf. Figure 8A) and thus make the spike trains more regular, the renewal approximation shows again some disagreement with the power spectrum of recurrent network neurons. The Gaussian approximation, on the contrary, yields once more the correct spectrum.

**Figure 9:**
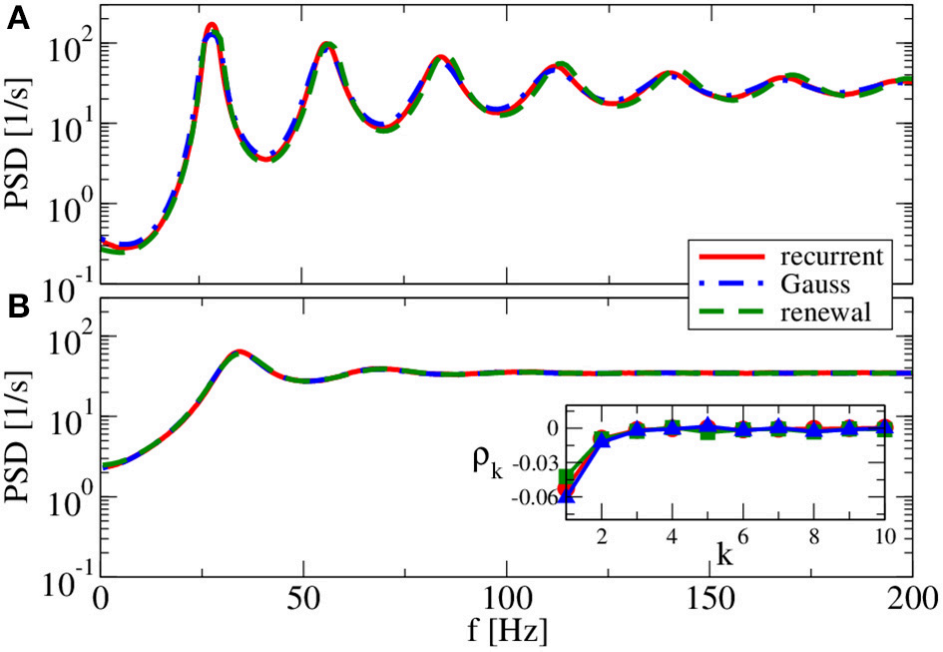
**Power spectra for dominating network inhibition**. Results of recurrent network simulations and the two approximations from our iterative schemes for *g* = 4.5 (inhibitory local synaptic input). **(A)** *v*_R_ = 0 mV (firing rate ν = 28.1 Hz, *C_V_* = 0.088); **(B)** *v*_R_ = 10 mV (firing rate ν = 34.8 Hz, *C_V_* = 0.27). Inset shows serial correlation coefficients for **(B)**.

For *g* = 4.5 (Figure 9), both renewal and Gaussian approximations agree with each other and with the network spectra, regardless of the value of the reset voltage. One might be tempted to think that this agreement is achieved because the spike-train statistics are closer to a renewal process. However, for this case we observe in both approximations as well as in the network simulations ISI correlations of the same order of magnitude as in Figure 7—only that correlations are negative in Figure 9, whereas they were positive in Figure 7.

What causes the failure of the renewal approximation in some of the cases considered above? Generally, it has become clear that the self-consistent noisy current stimulus is temporally structured. It is a colored noise, that in general leads to a non-renewal spike train of the driven neuron model (Middleton et al., 2003; Lindner, 2004). In particular, a narrow-band noise as we observed in Figures 7, 8A can lead to pronounced interval correlations (Bahar et al., 2001; Neiman and Russell, 2005; Bauermeister et al., 2013). Differences between the spectra in the recurrent network and that of our self-consistent renewal scheme are therefore not completely unexpected. Surprising is that the renewal scheme still works in some cases in which we saw pronounced ISI correlations.

**Figure 10:**
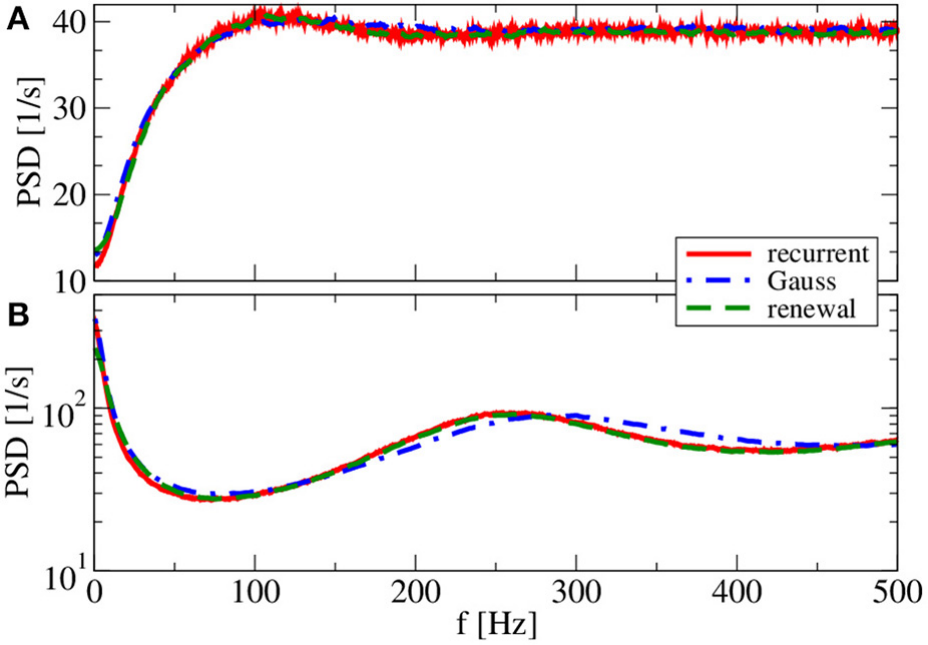
**Effect of larger synaptic amplitude on power spectra**. Results of recurrent network simulations and the two approximations from our iterative schemes for a synaptic amplitude of *J* = 1 mV, a smaller number of synapses *C*_E_ = 100, *C*_I_ = 25, and (*g* = 4.5) (inhibitory local synaptic input). **(A)** (*v*_R_ = 0 mV) (firing rate ν = 38.6 Hz, *C_V_* = 0.58); **(B)** (*v*_R_ = 10 mV) (firing rate ν = 65.7 Hz, *C_V_* = 1.98).

So far, the Gaussian approximation worked well for all chosen parameters inspected. The reason for this is the small amplitude of postsynaptic potentials (*J* = 0.1 mV) we have used in all simulations. We finally show and discuss a case with a larger amplitude (*J* = 1 mV in Figure 10). Because we do not want to change the mean input to the cell, we also reduce the number of synapses by a factor of 10. With a smaller number of synapses and a larger synaptic amplitude, we increase the noise in the system, which changes the shape of the power spectrum drastically, in particular, for the reset value of *v_R_* = 10 mV (Figure 10). Our main point with Figure 10, however, is that the renewal scheme in this case leads to a spectrum that is somewhat closer to the spectrum in the network than the Gaussian approximation for both values of the reset voltage. In this case, the assumption of the Gaussian approximation seems to be more severely violated than the assumptions of the renewal approximation are.

## 4. Discussion

The efforts in this paper were directed toward a better understanding of temporal correlations in recurrent neural networks. Here we focused on the simple case of a sparse homogeneous network, in which the autocorrelation of a single spike train is the only relevant temporal correlation of interest, i.e., cross-correlations between neurons can be neglected. To this end we introduced and compared two iterative simulation schemes, one of which is a simplified and numerically more efficient version of the framework put forward by Lerchner et al. (2006). Both simulation schemes correspond to an infinitely sparse network, because all input spike trains are completely independent of each other and only share the same statistics. In this way we escaped from the trap of complete synchronization, seen previously in finite layer-feedforward networks (Wang et al., 2006), which does not adequately describe the asynchronous state in a recurrent network.

Our results demonstrate in line with previous results by Lerchner et al. (2006) that the power spectrum of a single neuron in an unstructured network in the asynchronous state may be determined in some cases in a self-consistent approximation using iterative simulations of essentially *only one neuron*. We offer, to the best of our knowledge, the first comparison of such self-consistent spectral statistics with the respective statistics of the stationary state of the approximated LIF network. Moreover, we obtain strong numerical evidence from network simulations that these statistics do not vary with (a change of an uniform) synaptic delay as long as the latter yields an asynchronous state.

We showed that the iterative schemes (be it renewal or Gaussian one) do not work for too strong inhibition, because here instead of approaching a stationary spectrum, already the firing rate becomes unstable (an instability that is not present in the recurrent network), preventing a self-consistent determination of the spectrum. On the other hand, both schemes fail in any case for a non-sparse configuration (i.e., if *C*_E_/*N*_E_ ≪ 1 is not obeyed), because cross-correlations between input neurons cannot be neglected anymore and, hence, approaches solely based on single-neuron statistics cannot reproduce the correct power spectrum as measured in the recurrent network. Finally, our approach requires that even in the sparse network no synchronization emerges. This implies e.g., that we cannot reproduce the spike-train power spectrum for *g* < 3 (dominance of excitatory input coming from the network), for which strong synchronization is observed (Brunel, 2000). Preliminary simulation results for the recurrent network indicate that upon the transition to the synchronous regime, a peak at the population frequency arises in otherwise unchanged spectra.

In the important case of asynchronous activity close to the balanced state, the two methods to generate surrogate input work best in distinct limits. The Gaussian approximation can accurately predict the spike-train statistics of a neuron in the recurrent network as long as the synaptic amplitudes are sufficiently small. The renewal approximation in turn shows systematic deviations because of ISI correlations that are typical if a neuron is driven by a correlated noise. It may work better than the Gaussian approximation and the similar framework of Lerchner et al. (2006), however, if the amplitude is larger, e.g., for a value of *J* = 1 mV that is still within the physiological range (Koch, 1999). Here the renewal approximation performs better because it maintains the pulsatile nature of spike-train input (shot noise). Because it is known that the shot-noise character of synaptic input may affect firing rate and response properties substantially (Richardson and Swarbrick, 2010), this limit of larger amplitudes is worthwhile additional exploration. In particular, more elaborate generators for surrogate spike trains with prescribed second-order statistics (Brette, 2009) should be employed in this case.

From a more abstract point of view, our self-consistent scheme boils down to the question of finding an input stimulus, temporally correlated in such a way that it evokes in a non-linear neuron model a spike train with the very same temporal correlation. It is clear that without further constraints, this problem could have several solutions. Here we showed that under the special constraint of a Gaussian input statistics, the iterative scheme converges in a parameter regime close to the so-called balanced state to a unique second-order statistics. At the moment it is not clear how one could prove the existence and uniqueness of the solution mathematically.

The iterative scheme for the determination of self-consistent spectra can be extended in several directions. For the sake of comparison to the classical study by Brunel (2000), we used in this paper current-based synapses with an instantaneous spike input. Preliminary results show that the self-consistent determination can be applied to neurons with conductance-based instead of current-based input, including a first-order kinetics (low-pass filter) for the synapse. Another manageable extension would be to consider two populations of excitatory and inhibitory neurons with different synaptic and neural parameters, which in our framework would imply to simulate a single excitatory and a single inhibitory neuron receiving different inputs. Last but not least, it appears conceivable to determine the self-consistent cross-correlations between two neurons in iterative scheme(s) that employ simulations of *two uncoupled neurons* that receive correlated input characterized by the power and cross-spectra of the previous generation. Whether such a scheme can successfully reproduce the spike statistics may also depend on the specific connectivity and, in particular, on the amplitude of synaptic spikes, as it has been recently shown that in networks in which the number of presynaptic neurons scales with the network size, so-called spike echos additionally shape neural cross-correlations (Helias et al., 2013).

Our results can be regarded as a further step toward a more general theory of biological neural networks that takes the temporal structure of neural activity in the network more faithfully into account. Although in many instances, the Poissonian approximation may give a lot of insights and even a network of Poisson neurons may turn out to approximate the recurrent network reasonably well (Ostojic, 2014), there are also examples where exactly the temporal structure of spike trains matters (Câteau and Reyes, 2006). Helpful for analytical approaches would be formulas for the spectral spike-train statistics of simple IF models, which are driven by an Gaussian noise with an arbitrary (in particular, non-flat) power spectrum. Once a formula is known that provides the output spike-train power spectrum as a functional of the input power spectrum of the stimulating Gaussian noise, this functional could be regarded as a map, the fixed point(s) of which would yield the stationary solution(s) of our numerical procedure. So far, however, the problem of the spike statistics of a general IF model driven by arbitrary colored noise is an open issue in computational neuroscience. Most efforts have been focussed on the special case of low-pass filtered input noise (see e.g., Brunel and Sergi, 1998; Brunel et al., 2001; Brenner et al., 2002; Moreno-Bote and Parga, 2006; Alijani and Richardson, 2011) and only rarely more general forms of input correlations have been addressed analytically (Câteau and Reyes, 2006; Bauermeister et al., 2013).

Progress along these lines may nevertheless be possible, as a recent study by Schwalger et al. (submitted) illustrates: for the special case of a perfect IF model driven by a weak Gaussian noise with arbitrary input correlations, there exists a simple relation between the input correlation function (i.e., the Fourier transform of the input power spectrum) and the output spike-train statistics such as the ISI probability density and the interval correlations. Other approaches for IF neurons with threshold noise (Lindner et al., 2005a) or for threshold-crossing devices (Tchumatchenko et al., 2010) also permit approximations for the relation between spike-train power spectrum and colored Gaussian input noise. Results like these may be useful to calculate the self-consistent power spectrum in a recurrent network at least semi-analytically by finding the fixed point of the non-linear relation between input and output correlation functions.

### Conflict of interest statement

The authors declare that the research was conducted in the absence of any commercial or financial relationships that could be construed as a potential conflict of interest.

## Footnotes

^1^The resulting power spectrum of this first generation can be considered as the asymptotic spectrum resulting from an iterative scheme, in which we approximate the input as Poissonian spike trains that are solely determined by the output rate of the previous generation.
